# Galectin-1 Exerts Inhibitory Effects during DENV-1 Infection

**DOI:** 10.1371/journal.pone.0112474

**Published:** 2014-11-13

**Authors:** Karina Alves Toledo, Marise Lopes Fermino, Camillo del Cistia Andrade, Thalita Bachelli Riul, Renata Tomé Alves, Vanessa Danielle Menjon Muller, Raquel Rinaldi Russo, Sean R. Stowell, Richard D. Cummings, Victor Hugo Aquino, Marcelo Dias-Baruffi

**Affiliations:** 1 Department of Biological Sciences, Universidade Estadual Paulista – UNESP (FCL-Assis), Assis, Brazil; 2 Departmento de Análises Clínicas, Toxicológicas e Bromatológicas, Faculdade de Ciências Farmacêuticas de Ribeirão Preto, Universidade de São Paulo, Ribeirão Preto, Brazil; 3 Emory University School of Medicine, Atlanta, Georgia, United States of America; University of Texas Medical Branch, United States of America

## Abstract

Dengue virus (DENV) is an enveloped RNA virus that is mosquito-transmitted and can infect a variety of immune and non-immune cells. Response to infection ranges from asymptomatic disease to a severe disorder known as dengue hemorrhagic fever. Despite efforts to control the disease, there are no effective treatments or vaccines. In our search for new antiviral compounds to combat infection by dengue virus type 1 (DENV-1), we investigated the role of galectin-1, a widely-expressed mammalian lectin with functions in cell-pathogen interactions and immunoregulatory properties. We found that DENV-1 infection of cells in vitro exhibited caused decreased expression of Gal-1 in several different human cell lines, suggesting that loss of Gal-1 is associated with virus production. In test of this hypothesis we found that exogenous addition of human recombinant Gal-1 (hrGal-1) inhibits the virus production in the three different cell types. This inhibitory effect was dependent on hrGal-1 dimerization and required its carbohydrate recognition domain. Importantly, the inhibition was specific for hrGal-1, since no effect was observed using recombinant human galectin-3. Interestingly, we found that hrGal-1 directly binds to dengue virus and acts, at least in part, during the early stages of DENV-1 infection, by inhibiting viral adsorption and its internalization to target cells. To test the in vivo role of Gal-1 in DENV infection, Gal-1-deficient-mice were used to demonstrate that the expression of endogenous Galectin-1 contributes to resistance of macrophages to *in vitro*-infection with DENV-1 and it is also important to physiological susceptibility of mice to *in vivo* infection with DENV-1. These results provide novel insights into the functions of Gal-1 in resistance to DENV infection and suggest that Gal-1 should be explored as a potential antiviral compound.

## Introduction

Dengue is a mosquito-borne viral disease of expanding geographical range and incidence, it is estimated that up to 3.6 billion people live in endemic regions [reviewed in reference 1]. Recent estimates indicated that the number of infections worldwide is 400 million with ∼500,000 episodes of severe dengue, and <20,000 dengue related deaths per year [1].

Dengue is predominantly transmitted by the mosquito *Aedes agypti* and is caused by dengue viruses (DENV), a group of four serologically distinct positive strand RNA viruses: DENV-1, DENV-2, DENV-3, and DENV-4. They belong to the Flaviviridae family and genus Flavivirus (reviewed in [2]). Infection with any serotype can induce a range of disease from sub-clinical to a severe disorder. The severe disorder is associated with hemorrhage and plasma leakage which are recognized as dengue hemorrhagic fever (DHS) or dengue shock syndrome (DSS) [3], [4]. There are currently no specific treatments for dengue disease [5], and therefore, only supportive care is given [6]. Thus, antiviral compounds need to be identified in view of the spread of dengue disease throughout the world [5].

To identify control mechanisms for Dengue disease, we investigated the physiological functions of an endogenous innate immune protein named galectin-1 (Gal-1), a β-galactoside-binding lectin, in controlling infection caused by dengue virus (DENV-1). Galectin-1 is a ubiquitously expressed lectin, and can occur in both intracellular (cytoplasm and nucleus) as well as extracellular (cell surface and serum) compartments, despite the lack of a signal peptide for classical secretion [7]. Galectin-1 is differentially expressed by various normal and pathological tissues, including muscle, heart, liver, kidney, prostate, lymph nodes, spleen, thymus, placenta, testis, retina and also in immune and non-immune cells [8]. For instance, during infection or inflammation, Gal-1 may be released by infected epithelium, activated macrophages, and endothelial cells [8]. In fact, concerning endothelial cells, it has been extensively demonstrated that Gal-1 contributes to multiple steps of the angiogenesis cascade and then it has pro-angiogenic activity (reviewed in [9]).

Gal-1 exists in a monomer-dimer equilibrium, and in its dimeric form, the lectin can mediate cell-cell or host-pathogen interactions [10], [11], [12], similar to other members of the galectin family [13], [14] and other mammalian lectin families [15]. It has been extensively shown that it presents an immunomodulatory effect on microbial infections [16]. This lectin has a role in viral infections but its mechanisms and physiological functions are not clear. While some groups have reported an antiviral activity of Gal-1 during infections caused by Nipah virus [17], [18], Nodavirus [19], Influenza virus [20] and human simplex virus 1 (HSV-1) [21], other groups have reported that Gal-1 promotes infections caused by human immunodeficiency virus 1 (HIV-1) [22]-[27], HSV-1 [28] and human T-lymphotropic virus 1 HTLV-1 [29].

To our knowledge, the role of Gal-1 in DENV infection is yet to be evaluated. Here we show that both endogenous and exogenous Gal-1 inhibits DENV-1 infectivity, both in *in vitro* and *in vivo* infection in mice. Our results suggest that recombinant Gal-1 might have potential use as a novel approach to control DENV-1-induced pathology.

## Materials and Methods

### Cell lineages

The mosquito cell lineage from *Aedes albopictus* (C6/36) was cultivated at 28°C in L-15 medium (Leibovitz) (Cultilab, Campinas, Brazil) supplemented with 0.3% tryptose phosphate broth, 0.02% glutamine, 1% minimum essential medium (MEM) non-essential amino acids solution and 5% fetal bovine serum (Hyclone, Logan, USA). Vero-E6 (African green monkey kidney, ATCC CCL-81) cells were grown at 37°C in DMEM (Gibco, Life Technologies, Gaithersburg, MD, USA) supplemented with 10% fetal bovine serum. The human urinary bladder carcinoma cells (ECV-304, ATCC CRL-1998) were maintained at 37°C in RPMI-1640 medium (Gibco). Human lung microvascular endothelial cell lineage (HMVEC-L; ATCC CC2527) was cultivated in EC growth medium (EBM-2; Cambrex, Walkersville, MD, USA) containing 5% fetal bovine serum, human recombinant epidermal growth factor, human recombinant insulin-like growth factor-1, human basic fibroblast growth factor, vascular endothelial growth factor, hydrocortisone, ascorbic acid, gentamicin, and amphotericin B.

### DENV-1

The stock of the DENV-1 (strain Mochizuki, GenBank: AB074760.1) was prepared in C6/36 cells and titrated by plaque formation on Vero-E6 cells, as described previously [30]. Supernatants containing virus were collected and stored at −80°C for use in *in vitro* assays. *In vivo* assays were performed with the DENV-1 mouse brain-adapted strain, generated from Mochizuki strain in the same manner as described in reference [31]. Mouse-brain adapted DENV-1 was used on in vivo assays because non-adapted mouse strains do not readily replicate or cause pathology in immunocompetent mice, which is the case of mice used in the present study. Therefore, mouse-adapted DENV is lethal after intracranial challenge, and this severity parameter was used here to assess the physiological impact of Gal-1.

### Mice and survival study

Gal-1-deficient (*Lgals1^–/–^*) mice and age-matched wild-type (WT) mice on a C57Bl-6 background were used in experimental DENV-1 infections. For mortality assays, groups ranging from six to eight 3-day-old mice from both WT and *Lgals1^–/–^* lineages were infected with mouse-brain adapted DENV-1 as previously described, with modifications [31], [32]. Briefly, mice were intracerebrally injected with 10^6^ virions diluted in a total volume of 10 µl of phosphate buffer after anesthetization with a mixture of 10 mg/Kg of xylasine (Dopalen Vetbrands) and 100 mg/Kg of ketamine (Dopaser Hertap Caller). The brain macerates from non-infected mice were used for mock infection. The brains from non-infected and infected mice were collected after animals were euthanized by inhalation of carbon dioxide. Subsequent to the intracranial inoculation mice were examined daily, and mortality rates, as a direct result of the infection, were recorded up to 10 days post-infection. After this period, the surviving animals were euthanized by inhalation of carbon dioxide.

### Macrophage cultures

Resident macrophages were obtained from peritoneal washouts of 4–6 weeks-old WT and *Lgals1^–/–^* mice, after euthanasia by inhalation of carbon dioxide. Cells were suspended in RPMI-1640 medium supplemented with 10% of fetal bovine serum and allowed to attach onto 24-well plates. After an overnight incubation period, unattached cells were removed and adherent cells (macrophages) were used for *in vitro* infection with DENV-1 or submitted to Gal-1 expression analysis using Western Blot assay or conventional PCR methods.

### Ethical aspects

The present study uses death endpoint as a direct result from infection with DENV-1 and without humane euthanasia. This choice was strongly justified based on the following reasons: 1) the alternative use of humane endpoints based on clinical criteria was not possible since the earlier and reliable indicators of disease severity biomarkers had not been established or validated at the times the experiments were performed; 2) Our study was designed so that the survival assay consisted of a total of 35 mice from each lineage. If we had proposed to establish and validate biomarkers for severity, in order to use a humane endpoint, we would have had to use a much larger number of mice, which in our view would be unnecessary and raise other ethical concerns; 3) The mouse strain C57BL-6 was used in our study because its genetic counterpart, the *Lgals1^–/–^* mice, is naturally resistant to infection with DENV-1, so it was necessary to use a high inoculum in newborn mice, which promotes death during the acute infection; 4) All mouse experiments were performed under approved conditions in accordance to Faculdade de Ciências Farmacêuticas de Ribeirão Preto – Universidade de São Paulo (USP) Institutional Animal Care and User Committee approved protocols. The Ethics Committee on Animal Research of the University of São Paulo approved all the procedures described (Protocol Number: 10.1.1300.53.0).

### Human recombinant Galectin-1 (hrGal-1) and Galectin-3 (hrGal-3)

Recombinant forms (dimeric and monomeric) of human galectin-1 (hrGal-1) and human galectin-3 were prepared based on procedures previously described [33]–[36]. In addition, purified hrGal-1 was treated with 100 mM iodoacetamide (Sigma-Aldrich, MO, USA) in 100 mM lactose/PBS overnight at 4°C, as described [10], [18], [22], [33]. To ensure that hrGal-1 and hrGal-3 samples were endotoxin free, Detoxi-Gel Endotoxin removing gel (Pierce Biotechnology, Rockford, IL) was used. The activity of all produced galectins was assessed by hemagglutination. Biotinylation of hrGal-1 was performed using sulfo-NHS-LC-biotin (sulfosuccinimidyl 6-[biotinamido] hexanoate) (Pierce), according to the manufacturer's recommendations.

### In vitro viral infection

Indicated cell lineages were inoculated with DENV-1 (MOI 0.5) or vehicle solution (supernatant from non infected cells) and cultivated for 24, 48, 72, 96 or 120 hours, as indicated, at 37°C in a humidified, CO_2_-controlled atmosphere in appropriate medium supplemented with 10% FBS. Following, supernatants of culture were recovered for determination of viral load. Gal-1 effects were assessed by incubating cells, virus or both with the indicated concentrations of this lectin for 1 hour at 37°C before viral inoculum, as indicated. To evaluate the involvement of Gal-1 CRD in its antiviral activity the treatments were done in the presence of 10 mM lactose or sucrose.

### Adsorption and Internalization assays

Adsorption and internalization assays were performed as described previously [37] with modifications. For adsorption assays, ECV-304 cells were seeded in 24-well plates and infected with DENV-1 with a MOI of 10, in the presence or absence of 10 µM of hrGal-1, for 1 hour at 4°C. Following, cells were washed twice with PBS and immediately submitted to total RNA extraction or stored at −80°C until the time of use. For the internalization assays, cells were seeded in 24-well plates and inoculated with DENV-1 (MOI 10), for 1 hour at 4°C. After the incubation period, the inoculum was removed by 2 washes with PBS. The cells were then incubated with medium containing or not 10 µM of hrGal-1, for additional 1 hour at 37°C. The cells were then washed with PBS and treated with citrate buffer for 1 min to inactivate the adsorbed but not internalized virus. Finally, cells were washed with PBS to remove citrate buffer and stored at −80°C for subsequent quantification of viral load.

### Real-Time and conventional PCR

Viral loads were quantified in the culture supernatants using a one-step quantitative Real-Time PCR [38]. The total viral RNA purified from 1×10^7^ PFU of DENV-1 were 10-fold serially diluted to generate a standard curve. The viral RNA was purified using the QIAamp Viral RNA minikit (QIAGEN; Hamburg, Germany). Quantitative PCR reaction was carried out with the SuperScript III Platinum SYBR Green One-Step qRT-PCR kit (Invitrogen, Life Technologies) in a One-Step Real-Time PCR RT-PCR (Applied Biosystems, Life Technologies). Triplicate reactions were performed for each sample, and a no template control was included as a negative control. The primer sequences used for DENV-1 detection were RNC5-S: 5′-3′AGTTGTTAGTCTACGTGGACCGA and RNC5-C: 5′-3′CGCGTTTCAGCATATTGAAAG.

Qualitative analysis of Gal-1 mRNA expression on WT and *Lgals1^–/–^* cells was performed using conventional RT-PCR. Total RNA was purified using RNeasy Protect Mini Kit (QIAGEN) and cDNA obtained using M-MLV reverse transcriptase (1U) and oligo dT primers (both from Invitrogen, Life Technologies). PCR amplification was performed with primers for mouse Gal-1 (pFBNdgal-1: 5′-3′CGGATCCCATATGGCCTGTGGTCTG and pRHXgal-1: 5′-3′GCTCG AGAAGCTTTCACTCAAAGGCC) and β-actin (Fb-actin: 5′-3′CCCTAGGC ACCAGGGTGTGA and Rb-actin 5′-3′:GCCATGTTCAATGGGGTACTTC).

### Virucidal assay

The virucidal activity of hrGal-1 was assessed as described in reference [37], with modifications. Briefly, DENV-1 (2×10^5^ PFU) was incubated at 37°C for 1 hour in presence or absence of 10 µM of hrGal-1. The incubation was performed in presence of absence of RNAse A (150 mg/mL, Sigma-Aldrich). After one hour, the viral RNA was purified using QIAamp Viral RNA minikit and the samples were subjected to Real-Time PCR as described above. Purified viral RNA, treated or not with RNAse A, was used as positive control of reaction.

### Enzyme-linked Immunosorbent Assay

Soluble Gal-1 present in the culture supernatants was quantified by ELISA. Briefly, 96-well microplates were coated with rabbit polyclonal anti-Gal-1 antibody (1 µg/ml, produced in our laboratory). Plates were washed (PBS-Tween 0.05%) and incubated for 2 h at 37°C with blocking buffer (PBS/FBS 3%). Next, supernatant samples were added to plates and incubated at room temperature for 2 hours. After extensive washing, a chicken polyclonal anti-Gal-1 antibody (2 µg/ml; produced in our laboratory), diluted in PBS/FBS 3%, was added to each well and incubated for 1 h, at 37°C. After washing, wells were incubated with HRP-conjugated donkey anti-chicken IgY (Jackson ImmunoResearch, West Grove, PA, USA) for 1 h, at 37°C. Subsequently; substrate solution (substrate buffer, 1% TMB and 1% H_2_O_2_) was added. After a 20 min incubation period at room temperature, the reaction was stopped by addition of stop solution (5.5% H_2_SO_4_). Absorbance was determined at 450 nm using an ELISA reader (Thermo Labsystems, Franklin, MA, USA). A standard curve ranging from 20 to 20,000 pg/ml of hrGal-1 was generated for each ELISA.

The ability of hrGal-1 to bind DENV-1 was also tested by ELISA. Each well in 96-well microtiter plates was coated with 1 µg of hrGal-1 or 1% BSA overnight at 4°C. Plates were then rinsed once with PBS-Tween 0.05% and incubated with blocking buffer (PBS-Tween 0.05%, BSA 3%) for 2 hours at room temperature. After washing, serial two-fold dilutions of DENV-1 were added to each well and plates were incubated for 2 hours at room temperature. After three washes with PBS-Tween 0.05%, each well was incubated with mouse anti-E protein IgG (AbD Serotec, Raleigh, NC, USA) for 1 h, at 37°C. Following this incubation step, plates were washed and incubated with HRP-conjugated donkey anti-mouse IgG (Jackson IR) for 1 h, at 37°C. The development of peroxidase reaction was performed with TMB substrate, as describe above. To assess the participation of Gal-1 CRD on viral-lectin interactions, different concentrations of lactose or sucrose were added to wells before the addition of virus dilutions.

### Flow cytometry

Measurement of free ligands for Gal-1 on ECV-304 cells surface was performed by incubating ECV-304 cells with 10 µM biotinylated-hrGal-1 for 1 hour at 4°C, in presence or absence of 40 mM lactose or sucrose (Sigma-Aldrich). After washing, cells were incubated with streptavidin-FITC (Jackson IR) for 30 minutes at 4°C, washed and fixed. Labeled cells were acquired on a FACS Canto (Becton Dickinson, Mountain View, CA, USA) and analyzed in the DIVA software (Becton Dickinson).

### Cell death assay

Apoptosis and necrosis signals were investigated through propidium iodide (PI) and Annexin-V staining. Membrane permeability was evaluated in fresh ECV-304 cells, after immediate addition of propidium iodide (2 mg). DNA degradation was detected in ECV-304 cells gently resuspended in 0.3 ml hypotonic PI solution (PI, 50 µg/ml in 0.1% sodium citrate plus 0.1% Triton X-100; Sigma-Aldrich). Tubes were kept at 4°C for 16 hours in the dark. Cells treated with Camptothecin (CPT – 10 µM – Sigma-Aldrich) were used as positive inducer of cellular death.

### Cell viability assay

Cell viability was measured by the colorimetric MTT (1-(4,5-Dimethylthiazol-2-yl)-3,5-diphenylformazan, Sigma-Aldrich) assay as previously described [39].

### Western blot assay

Cells were lysed in sample buffer (62.5 M Tris, pH 6.8, 2% SDS (w/v), 5% glycerol (v/v), 30 µM phenol red and 0.9% β-mercaptoethanol) and incubated for 5 min at 100°C. Samples were resolved in 15% polyacrylamide gels and transferred onto nitrocellulose membranes (Amersham Biosciences, Uppsala, Sweden). After saturation with 5% non-fat dry milk, membranes were probed with mouse monoclonal anti-Gal-1 or mouse monoclonal anti-β-actin (Abcam, MA, EUA) for 2 hours at room temperature. After washing, membranes were incubated with HRP-conjugated donkey anti-mouse IgG (Jackson IR) for 45 minutes at room temperature. Bound antibodies were revealed by enhanced chemiluminescence using the ECL kit (Pierce).

### Data Analysis

Statistically significant differences among groups were assayed using analysis of variance (ANOVA) (Bonferroni Dunn test). Values of *p*<0.05 were considered significant results.

## Results

### Reduced expression of endogenous Gal-1 results in increased permissiveness to DENV-1 infection

**Figure 1:**
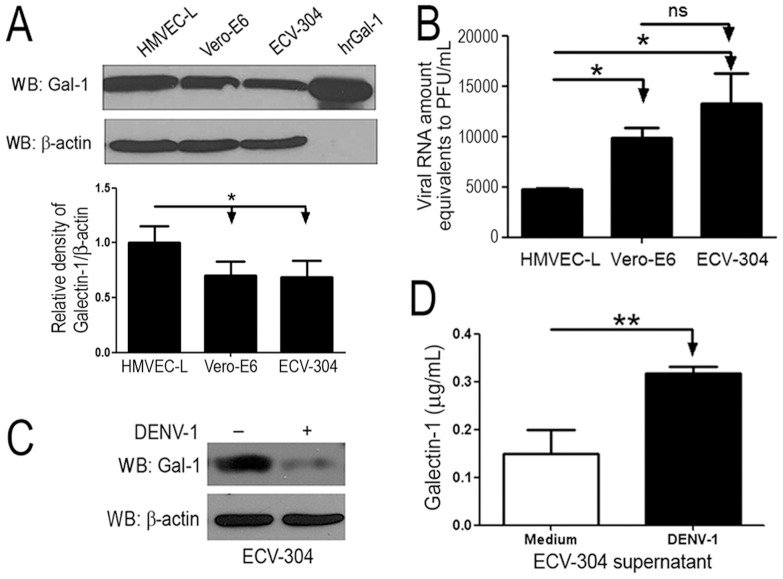
Lower expression of Gal-1 is correlated with higher viral loads produced by DENV-1-infected cells. **(A)** Gal-1 expression on HMVEC-L, Vero-E6 and ECV-304 cells was assessed by western blot method and normalized by β-actin endogenous control. The relative density of Gal-1 was determined by ImageJ software. **(B)** HMVEC-L, Vero-E6 and ECV-304 cells (2.5x10^4^) were incubated with DENV-1 (MOI 0.5) for 72 hours at 37°C. At the end of incubation period, the total amounts of viral RNA in the cell-free supernatants were determined by Real-Time PCR, using a standard curve constructed from DENV-1 RNA purified from 1x10^7^ PFU (PFU: plate formed units). Results are shown as Viral RNA amount equivalents to PFU/ml±SD from 3 independent assays performed in triplicates. **(C)** ECV-304 cells were inoculated with DENV-1 (MOI 0.5) or only with medium and cultivated for 72 hours at 37°C. Cells were analyzed for Gal-1 expression by western blot assay. **(D)** Soluble Gal-1 was detected in the supernatants from cell cultures using ELISA method (N = 3). *p<0.01; **p<0.001.

To investigate the role of endogenous Gal-1 in the course of DENV infection, we infected three different cell lines with DENV. Vero-E6 cells are known to be permissive to all four dengue virus serotypes [40]–[42], while ECV-304 (a carcinoma cell lineage with endothelial characteristics) and lung microvascular endothelial cells (HMVEC-L) are known to be permissive to dengue virus serotype-2 [24], [43]–[45]. Interestingly, ECV-304 and Vero-E6 cells express similar levels of Gal-1 protein, while HMVEC-L expressed somewhat higher levels of Gal-1 when compared to other cell lines tested (Figure 1A) and quantified by ImageJ program (Figure 1A). Next, cells were inoculated with DENV-1 (MOI 0.5) and cultivated for 72 hours, and the viral load was quantified from the culture supernatants by One Step Real-Time PCR. We found that Vero-E6 cells, which displayed the lowest Gal-1 expression level (Figure 1A), were highly permissive to DENV-1 infection (Figure 1B). In sharp contrast, we detected the lowest level of viral load in the supernatants of HMVEC-L cells (Figure 1B), which displayed the highest Gal-1 expression level (Figure 1A). To further investigate the relationship between Gal-1 levels and permissiveness to DENV infection, we infected the ECV-304 cells with DENV-1 for 72 h and evaluated Gal-1 expression by western blot. As shown in Figure 1C, the presence of DENV-1 decreased the expression of endogenous Gal-1 in comparison to that cells maintained in medium alone. In addition, ECV-304-infected cells secreted more Gal-1 to culture supernatants (Figure 1D), which could explain at least in part the reduction of cell-associated Gal-1 protein after infection (Figure 1C).

### Treatment with recombinant hrGal-1 reduces viral production in DENV-1 virus-infected cells

**Figure 2:**
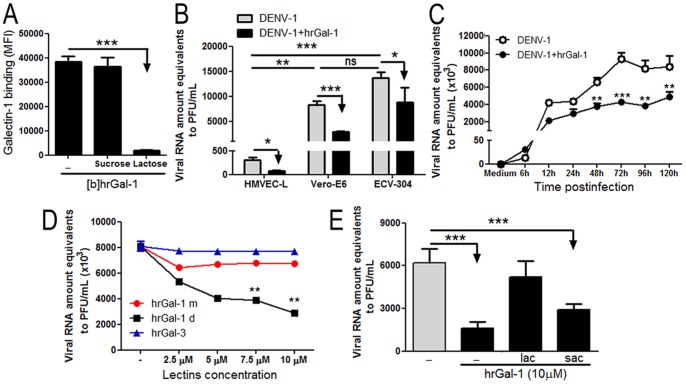
Treatment with human recombinant Gal-1 inhibits DENV-1 in vitro infection. **(A)** Biotinylated-hrGal-1 (20 µg/mL) was incubated with ECV-304 cells in presence or absence of 40 mM lactose or sucrose for 1 hour at 4°C. The binding of biotinylated-hrGal-1 to ECV-304 cells surfaces was detected by staining with streptavidin-FITC and measured by flow cytometry. The analysis was performed using a Diva software (Becton Dickson) and results are expressed as mean mean fluorescence intensity (MFI)±SD. Tests were performed in triplicates. **(B)** HMVEC-L, Vero-E6 and ECV-304 cells were incubated with 10 µM hrGal-1 or only medium for 1 hour at 37°C. Following, cells were inoculated with DENV-1 at a MOI of 0.5 and cultivated for 72 hours. At 72 hours postinfection the supernatants were collected and the viral RNA amounts were quantified by Real-Time PCR. Results are shown as Viral RNA amounts equivalent to PFU/ml±SD from 3 assays performed in triplicates. **(C)** ECV-304 cells were treated with hrGal-1 and infected with DENV-1 as described in (B) for 120 hours. The supernatants were collected at the indicated times postinfection and the viral loads were quantified by Real-Time PCR as described in (B). (N = 3) **(D)** ECV-304 cells were incubated with increased concentrations of monomeric-Gal-1 (hrGal-1 m), dimeric-Gal-1 (hrGal-1 d) or galectin-3 (hrGal-3) for 1 hour at 37°C before inoculation with DENV-1 (MOI 0.5). Cells were cultivated for 72 hours at 37°C and viral load was quantified as described in (A) (N = 3). **(E)** ECV-304 cells were treated with hrGal-1 (10 µM) in the presence of 40 mM lactose (LAC) or 40 mM sucrose (SUC) and then infected with DENV-1 as described in (B), for 72 hours. The viral load was quantified as described in (B). Data are representative from three independent experiments. *p<0.01; **p<0.001; ***p<0.0001.

Since Gal-1 expression levels seemed to be inversely correlated to DENV permissiveness, we therefore evaluate whether the addition of exogenous Gal-1 could interfere with DENV infection in vitro. First, we demonstrated that ECV-304 cells display Gal-1-specific binding sites because human recombinant Gal-1 (hrGal-1) was able to bind ECV-304 cell surfaces, and this binding is abrogated in the presence of lactose, a weak but effective inhibitor of Gal-1, but not by sucrose, an isomeric sugar that does not bind galectins (Figure 2A). Similar results were obtained using HMVEC-L and Vero-E6 cells (data not shown). Monolayers of ECV-304, Vero-E6 and HMVEC-L cells were treated with 10 µM of hrGal-1 for 1 hour at 37°C, followed by addition DENV-1 virus (MOI 0.5) for 72 hours, and the viral load was quantified from the culture supernatants. As demonstrated before (Figure 1B), EVC-304 and Vero-E6 cells are much more permissive to DENV-1 infection than HMVEC-L cells; however, all the three cell lines had a significant reduction in viral load (35% in Vero-E6 cells, 60% in ECV-304 cells and 65% HMVEC-L cells) when pre-treated with hrGal-1 (Figure 2B). The kinetics of DENV-1 infection was also monitored in EVC-304 cells, pretreated or not with hrGal-1, during a period of 120 hours after infection. As shown in (Figure 2C), at 48, 72, 96 and 120 hours postinfection, the viral loads detected in the supernatant of DENV-1-infected ECV-304 cells pre-treated with hrGal-1 were significantly lower compared with viral load recovered from the supernatants of Gal-1-untreated cells. We also found a dose-dependent inhibition of DENV-1 infection when ECV-304 cells were pretreated with increasing hrGal-1 concentrations for 1 hour before the infection (Figure 2D).

It has been shown that Gal-1 exists in a monomer-dimer equilibrium, and several important functions of this protein have been shown to be dependent on Gal-1 dimerization [8], [36]. Therefore, we tested whether the inhibitory effect of Gal-1 on DENV-1 infection was dependent on its capacity to form dimers. Using a mutant Gal-1, which is unable to form dimers, we showed that the monomeric form of hrGal-1(m) had no capacity to reduce DENV-1 viral loads in the supernatant of EVC-304-infected cells compared with the hrGal-1 (d), indicating that dimerization is important for the inhibitor effects of Gal-1 on DENV-1 infection (Figure 2D). Importantly, we observed that this inhibitory effect was specific for hrGal-1 since galectin-3, another galectin binding protein from galectin family, and one which naturally occurs in multimeric forms, showed no inhibitory effect on vital load (Figure 2D).

To assess the involvement of the carbohydrate-recognition domain (CRD) of Gal-1 in this inhibitory effect, ECV-304 cells were pre-treated or not with hrGal-1 in presence or absence of a specific sugar inhibitor (lactose). Lactose blocked the inhibitory effect of hrGal-1 on DENV-1 infection, whereas sucrose did not block (Figure 2E), indicating a dependence of the Gal-1 CRD for these functions.

### Inhibitory effect of Gal-1 on DENV-1 release is not associated with induction of cell death

**Figure 3:**
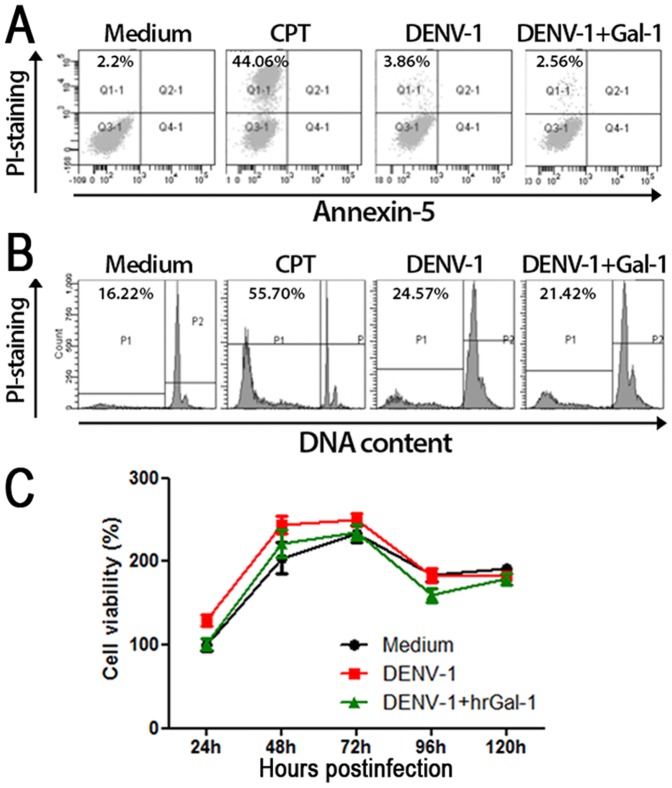
Gal-1 does not induce cell death during DENV-1 infection. **(A and B)** ECV-304 cells (2.5x10^4^) were treated with hrGal-1 (10 µM) and infected with DENV-1 (MOI 0.5). At 72 hours postinfection, PI staining was used to evaluate the membrane permeability (A) and DNA degradation (B). For these purposes, PI was added to fresh cells or to permeabilized cells, respectively. Samples were acquired and analyzed by flow cytometry. Data is representative from three experiments with similar results. **(C)** ECV-304 cells were treated or not with 10 µM of hrGal-1 and infected with DENV-1 as described in (A). At the indicated times postinfection, cell viability was examined by MTT assay. Cells cultivated only with medium at 24 hours were designated as 100% of cell viability. Data is representative of 3 independent experiments performed in triplicates.

We explored whether the antiviral effect of Gal-1 could be due to induction of cell death. Thus, we evaluated the apoptosis and necrosis of ECV-304 cells pre-treated or not with hrGal-1 and infected with DENV-1 virus. As can be seen in the Figure 3A, DENV-1-infected ECV-304 cells (pretreated or not with hrGal-1) did not show significant staining with either PI or Annexin-V-FITC, in contrast to cells treated with camptothecin, a positive inducer of cell death. In order to confirm this result, we also checked the degradation of chromosomal DNA and cell viability by using the MTT assay. Corroborating our previous result using Annexin-V/PI staining, pretreatment of cells with hrGal-1 before DENV-1 infection did not cause any effect on DNA degradation and cell viability (Figure 3B and C, respectively). Cell viability and apoptosis assays performed in Vero-E6 and HMVEC-L cell lineages infected with DENV-1 in presence or absence of hrGal-1 provided similar results (data not shown). Altogether, these data indicate that Gal-1 inhibits DENV-1 infection without inducing cell death in infected cells.

### hrGal-1 binds to DENV-1 and inhibits its adsorption and internalization processes in ECV-304 cells

**Figure 4:**
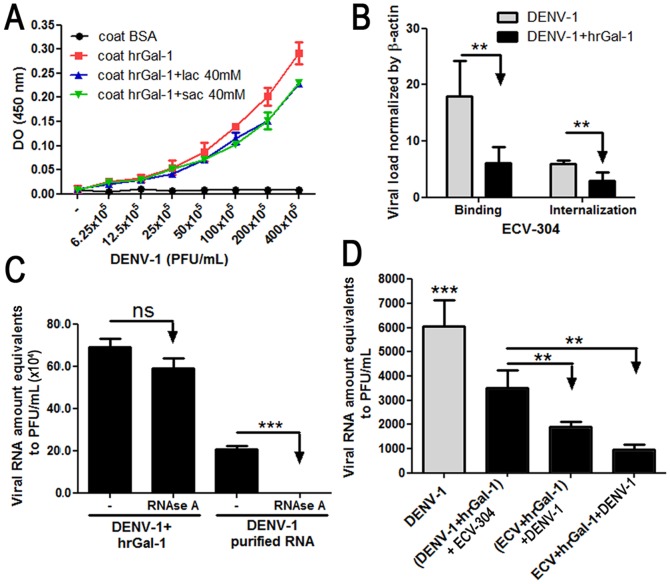
Gal-1 acts at early stages during DENV-1 infection. **(A)** Binding of DENV-1 to hrGal-1 in a dose-dependent manner. Serial two-fold dilutions of DENV-1 were applied to 96-well plates coated with 1 µg of hrGal-1 per well, and the bound virus particles were detected by ELISA with mouse anti-E protein antibody. BSA-coated wells served as the negative control. To assess the involvement of Gal-1 CRD, the assay was performed in presence of 40 mM lactose or sucrose. Each value represents the mean±the SD from 4 assays performed in duplicates. **(B)** Adsorption and internalization assays: for adsorption assay, ECV-304 cells were infected with DENV-1 at MOI of 10 in presence or absence of 10 µM hrGal-1 during 1 h at 4°C and then washed to remove viral inoculum. Cells were collected and the viral RNA was quantified by Real-Time PCR. Data was normalized by host β-actin expression. For internalization assay, ECV-304 cells were inoculated with DENV-1 (MOI of 10) at 4°C for 1 hour. Then, cells were washed and transferred to 37°C and hrGal-1 (10 µM) or only medium were added to culture. After 1 hour of incubation, non-internalized viruses were inactivated with citrate buffer and viral loads were quantified by Real-Time PCR. Data is presented as Viral RNA amount equivalents to PFU/mL±SD from 3 experiments assessed in triplicates. **(C)** For virucidal assay, DENV-1 was incubated with hrGal-1, in the presence or absence of RNAse. After 1 h incubation at 37°C, RNA was isolated and subjected to RT-Real-Time PCR. Purified viral RNA incubated or not with RNAse was used as control (N = 3). **(D)** ECV-304 cells were infected with DENV-1 at a MOI of 0.5 (DENV-1). For the treatments, hrGal-1 was incubated with ECV-304 and DENV-1 simultaneously (ECV+Gal-1+DENV), or hrGal-1 (10 µM) was pre-incubated with either ECV-304 cells or with DENV-1 (MOI 0.5) for 60 minutes before the inoculation (ECV+hrGal-1)+DENV versus (DENV+hrGal-1)+ECV, respectively. At 72 hours postinfection, supernatants were collected and the viral loads were quantified by Real-Time PCR (N = 3). **p<0.001; ***p<0.0001.

To further investigate the inhibitory effect of exogenous Gal-1 on DENV-1 infection, we first checked whether Gal-1 was able to bind directly to the virus and exert any *direct virucidal* activity against DENV-1. As shown in Figure 4A, DENV-1 bound to immobilized hrGal-1 in a dose-dependent manner, whereas the virus failed to bind to BSA-coated wells. Unexpectedly, the binding of DENV-1 to hrGal-1 was not abolished by the addition of lactose, an inhibitor of carbohydrate binding (Figure 4A). This may mean that the affinity of binding between hrGal-1 and DENV-1 is extremely high toward immobilized hrGal-1, and thus not readily reversible by lactose, or binding occurs through a non-carbohydrate interaction to hrGal-1. We next evaluated whether hrGal-1 could affect virus adsorption and/or virus internalization. As shown in Figure 4B, hrGal-1 significantly inhibited virus adsorption at 4°C for 1 hour (Figure 4B) when hrGal-1 was present during the incubation. If adsorption took place in a hrGal-1-free medium, and the lectin was added at culture supernatants when the temperature was raised at 37°C and maintained only additional 1 h of incubation, the virus yield also significantly decreased in comparison with untreated cultures (Figure 4B). A possible virucidal effect of hrGal-1 on DENV-1 was discarded by a virucidal assay (Figure 4C). Altogether, these results suggest that hrGal-1 may influence the early steps of DENV-1 infection. Finally, we investigated whether the inhibitory effects exerted by Gal-1 also depended on its interaction with the target cells. The pretreatment of DENV-1 with hrGal-1 [(DENV1+rhGal-1)+ECV] led to a significant decrease in viral load in the culture supernatants (Figure 4D). However, when ECV-304 cells were treated with hrGal-1 before DENV infection [(ECV+rhGal-1)+DENV1] or ECV-304 cells were treated concomitantly with hrGal-1 and DENV-1 (ECV+rhGal-1+DENV1), we observed an even greater reduction in the viral load in the culture supernatants (Figure 4D).

### Absence of endogenous Gal-1 leads to early mortality of mice to DENV-1 infection

**Figure 5:**
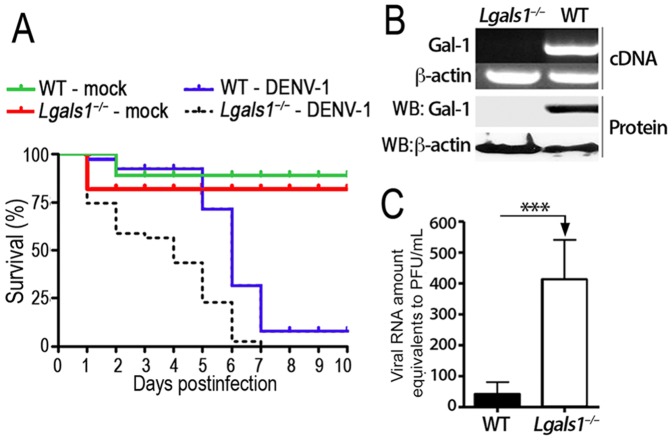
Gal-1 is physiologically relevant to in vivo infection with DENV-1. **(A)** Newborn WT and *Lgals1^–/–^* mice were intracerebraly infected with 10^6^ PFU/ml of DENV-1 mouse-brain adapted or mock (supernatant from mouse brain not infected with DENV-1) and their mortality was monitored for 10 days. Results are shown as the percentage survival from five independent assays performed with 6–8 mice per group. **(B)** Total mRNA isolated from resident peritoneal macrophages of Gal-1-deficient (*Lgals1^–/–^*) and wild-type (WT) mice was converted into cDNA and the Gal-1 expression was analyzed using conventional PCR. The β-actin gene was used as an endogenous control. Alternatively, total protein from *Lgals1^–/–^* and WT macrophages were isolated and Gal-1 expression was quantified by western blot assay and normalized to β-actin expression. **(C)** Peritoneal resident macrophages from *Lgals1^–/–^* or WT mice were cultivated (5x10^5^/well) in 24-well plates and inoculated with DENV-1 at a MOI of 0.5. After 72 hours at 37°C, cell-free supernatants were recovered and the viral loads were determined by Real-Time PCR. Results are showed as Viral RNA amounts equivalent to PFU/ml±SD from three experiments assessed in triplicates. ***p<0.0001.

Based on the results presented so far and to better demonstrate the role of Gal-1 in controlling DENV-1 infection, we next infected newborn Gal-1-deficient (*Lgals1^–/–^*) and wild-type mice with mouse-brain adapted DENV-1 virus and analyzed host survival for 10 days (Figure 5A). Although mice from both lineages displayed similar overall mortality rate, *Lgals1^–/–^* mice began to succumb earlier than WT mice: within the first 4 days post-infection, almost 60% of *Lgals1^–/–^* mice died, while 90% of WT mice survived up to 5 days past challenge (Figure 5A).

Since macrophages are important target for DENV virus in mouse models of dengue infection [46], [47] and we demonstrated above that there is an inverse correlation between DENV permissiveness and Gal-1 expression level, we evaluate the susceptibility of macrophages isolated from *Lgals1^–/–^* to DENV infection. As expected, macrophages from *Lgals1^–/–^* mice did not express Gal-1 mRNA or protein levels (Figure 5B). Corroborating the data obtained with endothelial cells (Figure 1A and 1B), we detected much higher viral load in the supernatant of *Lgals1^–/–^* macrophages than in the supernatants from WT macrophages (Figure 5C). This result suggests that endogenous Gal-1 contributes to mice resistance during infection with DENV-1.

## Discussion

Here we demonstrated that both endogenous and exogenous Gal-1 reduces DENV-1 infection by inhibiting virus infection of mammalian cells. Our results show that, at least in part, the inhibition by Gal-1 involves the prevention of adsorption and internalization of dengue virus into target cells. Also, the inhibitory effect of Gal-1 on DENV-1 infection depends on carbohydrate recognition and Gal-1 dimerization.

Gal-1 is widely expressed in animal cells and tissues; it is expressed in the thymus and by lymphoid parenchymal epithelial cells, endothelial cells, trophoblasts, activated T and B cells, macrophages, follicular DCs, and CD4^+^CD25^+^ regulatory T cells [12]. Gal-1 has an important immunomodulatory activity, playing essential roles during microbial infection by modulating both innate and adaptive immunity [16]. However, the role of Gal-1 in the context of viral infections is less clear. Gal-1 has an anti-viral effect on infections by Nipah virus [17], [18], Nodavirus [19], Influenza virus [20] and HSV-1 [21]. In such cases, Gal-1 negatively controls the infection by both directly interacting with viral glycoproteins, and thus inhibiting their mobility, maturation and functions [18], [40], and by affecting the immune response of the target cells after infection [17], [19]. However, Gal-1 can also promote the infectivity of cells by HSV-1 [28], HIV-1 [22]–[27] and HTLV-1 [29]. In such cases Gal-1 acts as a soluble adhesion molecule that stabilizes virus attachment to host cells and facilitates their entry [20]. Concerning DENV, there are no data in the literature exploring the role of Gal-1 in the infection caused by any of the four DENV-serotypes.

The initial steps leading to DENV entry into the host cells for primary infection are very poorly understood. Here we demonstrated that ECV-304, Vero-E6 and HMVEC-L cell lineages and WT murine macrophages are permissive to DENV-1 infection, and all of them constitutively express Gal-1 (Figure 1A and 5B). However, Gal-1 expression on HMVEC-L cells is higher when compared with the other two cell lineages, and interestingly, the permissiveness of HMVEC-L cells to DENV-1 is lower than ECV-304 and Vero-E6 cells. On the other hand, macrophages with no expression of Gal-1 (*Lgals1^–/–^*) are more permissive to DENV-1 infection. These data suggest an inverse correlation between Gal-1 expression and permissiveness to DENV-1 infection. It has been shown that dengue virus can interact with a large number of proteins [48], including the heat-shock protein 70 and DC-SIGN (a C-type mannose-binding lectin) [49]. It is also known that the galectins may form glycoprotein lattices on the cell surface [8], [23], directly affecting their distribution, functions and endocytosis [18]. Since HMVEC-L cells present higher expression of Gal-1 compared with ECV-304 and Vero-E6, it is tempting to speculate that the membrane-associated Gal-1 may interact with DENV glycoprotein E to form lattices, and thus inhibit DENV-1 infectivity by reducing the entry of virus particles. It is also possible that Gal-1 could inhibit subsequent virus maturation, similar to the observed for Nipah virus [18].

Although we can not rule out that other factors could be also affecting HMVEC-L susceptibility, the possibility that Gal-1 might restricts viral entry is corroborated by our findings, showing that addition of exogenous Gal-1 inhibits virus adsorption to ECV-304 cells and its internalization during *in vitro* infection (Figure 4B). This inhibitory effect is reflected on the decreased virus release at 72 hours postinfection, and it is observed not only in ECV-304 cells, but also in Vero-E6 and HMVEC-L cell lineages (Figure 2B). In this set of experiments we demonstrated that the inhibitory effect of Gal-1 on DENV-1 release depends on protein dimerization, previously demonstrated to be required for efficient cross-linking of functional receptors or for the formation of signaling lattices [50]–[52]. Also, hrGal-1 inhibitory effect was dependent on carbohydrate recognition as is specifically impaired by the presence of lactose (Figure 2E). Together, these findings support the idea that the inhibitory effect of Gal-1 partly involves extracellular activities of this protein, which may include the lattice-formation (which is CRD-dependent) and interferes with virus adsorption and internalization.

Gal-1 may also exert an inhibitory effect on DENV-1 release by affecting the host cell responses. It has been reported that Gal-1 expression is altered after viral infections (*Helicobacter pylori* [53] e HTLV-1 [29]. We found decreased cellular Gal-1 expression accompanied of accentuated levels of Gal-1 in the supernatants from DENV1-infected ECV-304 cells at 72 hours postinfection (Figure 1C and 1D). It is possible that once released to the extracellular milieu, Gal-1 could act as an autocrine regulatory factor for endothelial cells to limit viral spread or act as potential damage-associated molecular pattern (DAMP) [54], [55], thus interfering with the immune responses arising after DENV-1 infection.

It has been demonstrated that intracranial infection of mice on a C57Bl-6 background results in neurological abnormalities and death, but these mice does not show the most usual clinical signs observed in humans [30]–[33]. However, although we can not extrapolate our data concerning the impact of Gal-1 in in vivo DENV-1 infection of mice to DENV-1 infection in humans, this approach allowed us to develop insights into the mechanisms behind Gal-1 effects.

The lethality of mice infected with DENV-1 is also associated with increased vascular permeability induced by an uncontrolled release of pro-inflammatory cytokines known as “Cytokine storm” [49], [56]. Herein we demonstrated that newborn *Lgals1^–/–^* mice infected with DENV-1 start dying earlier than WT mice. This condition seems to be associated with the evidence that the *Lgals1^–/–^* macrophages (and probably other cell types as well) release higher amounts of virus particles at 72 hours post-infection, compared with the WT macrophages. Interestingly, there is uncertainty in the scientific community concerning DENV infectivity on macrophages. Some groups have noted that human and mouse macrophages are major cellular targets for DENV infection [46], [49], [57], [58], but others suggests that the virus does not efficiently infect these cells in the absence of sub-neutralizing antibodies [59]. In our system, macrophages from WT mice were infected with DENV-1, however, the viral load from these cells was very low (viral RNA amount equivalent to 50 PFU/mL, Figure 5C), compared to viral load from other cell lineages (between 5,000 and 20,000 PFU/mL, Figure 1B). Nevertheless, the viral load in macrophages from *Lgals1^–/–^* animals was about eight times higher than that from WT macrophages, suggesting that endogenous Gal-1 contributes to resistance of mice to DENV-1 infection. The absence of Gal-1 in newborn *Lgals1^–/–^* mice may favor the establishment of a stronger cytokine storm in these mice later in the infection, since Gal-1 is classically known as an important anti-inflammatory factor [8], [60]. Altogether, these conditions may contribute to the faster mortality observed in *Lgals1^–/–^* mice. Our results are in accordance to previous report showing that *Lgals1^–/–^* mice were also more susceptible to influenza virus infection compared with their WT counterparts [20].

Despite the increasing incidence of DENV as a human pathogen, there are no antiviral agents or vaccines for treatment or prevention [61]. Data presented here show that both endogenous and exogenous Gal-1 are inhibitory to DENV-1 infection. The differences between dengue pathogenesis in mouse and humans should be taken into account, but our results raise the possibility of using recombinant Gal-1 of as an additional/alternative method of treatment for dengue disease. This concept has also been advanced by others for the development of therapeutic treatments for pathogenic and non-pathogenic diseases [8], [62]–[64]. It has been shown that prophylactic or therapeutic administration of Gal-1 in animal experimental models of inflammatory diseases; cancer or neurodegeneration can ameliorate the disease symptoms or even the mice survival (reviewed in [8]). In the case of pathogenic diseases, the effects of recombinant Gal-1 administration are not well defined and have been shown to be context-dependent, since it can restrict or facilitate the infection [18], [22], [65]. Despite the difficulties in this field, the potential use of galectins as therapeutic targets has advanced. In the present work, we have shown that Gal-1 may interfere with the course of dengue virus infection probably through several mechanisms, including its participation in DENV-1 entry and cellular responses. Our future investigations aimed to elucidate the molecular mechanisms behind Gal-1 effects and also its roles during dengue human pathogenesis. Together, our study may promote the development of new drugs to combat the pathogenesis caused by this virus.
